# Antitumor Activity of a Synthetic Small RNA Based on a *Maytenus hookeri* Sequence in Non-Small Cell Lung Cancer

**DOI:** 10.3390/ijms27177601

**Published:** 2026-08-25

**Authors:** Dengyuan Liu, Xinmeng Yang, Sifen Du, Na Sun, Yexuan Lin, Yixin Dong, Chengyu Jiang

**Affiliations:** State Key Laboratory of Common Mechanism Research for Major Diseases, Department of Biochemistry, Institute of Basic Medical Sciences & School of Basic Medicine, Chinese Academy of Medical Sciences & Peking Union Medical College, Beijing 100005, China; dengyuanliu@ibms.pumc.edu.cn (D.L.); yangxinmeng1225@163.com (X.Y.); dusf1998@163.com (S.D.); sn1635093846@163.com (N.S.); linyexuan@bsjpharma.com (Y.L.); 18813045400@163.com (Y.D.)

**Keywords:** non-small cell lung cancer, *Maytenus hookeri Loes.*, small RNA, *KRAS*, oligonucleotide therapeutics

## Abstract

Lung cancer remains the leading cause of cancer-related mortality worldwide, highlighting the urgent need for novel therapeutic strategies. This study aimed to identify bioactive small RNAs derived from *Maytenus hookeri Loes.* and evaluate their anti-tumor potential against non-small cell lung cancer (NSCLC). The 50 most abundant small RNAs were synthesized and screened in NCI-H460 cells using the MTS assay. Among them, MDM-sRNA-39 significantly inhibited cell viability and was selected for further evaluation of its anti-tumor activity. It reduced the percentage of cells that traversed the Transwell membrane and promoted apoptosis in NCI-H460 cells. Bioinformatics prediction suggested *KRAS* as a potential target of MDM-sRNA-39, and *KRAS* was among the targets experimentally validated by dual-luciferase reporter assays, RT-qPCR, and Western blot, showing that MDM-sRNA-39 suppresses *KRAS* expression at both mRNA and protein levels. *In vivo*, MDM-sRNA-39 was orally administered as a bencaosome in a *Kras^LSL-G12D^/p53^LoxP/LoxP^* autochthonous lung cancer mouse model. Tumor burden was reduced and cleaved caspase-3 staining was increased in the mouse model. Collectively, these findings identify MDM-sRNA-39, a synthetic small RNA based on a sequence identified from *Maytenus hookeri Loes.*, as a candidate with anti-tumor activity, supporting the potential strategy for developing RNA-based therapeutics from traditional Chinese medicine.

## 1. Introduction

NSCLC) represents for the most common of lung cancer cases and continues to be a primary contributor of cancer-related mortality worldwide [1]. Although surgery, chemotherapy, radiotherapy, immunotherapy, and molecular targeted therapies [2,3] have improved clinical outcomes, many patients still experience disease progression, treatment-related toxicity, or therapeutic resistance [4]. These limitations support the continued search for new therapeutic strategies with distinct mechanisms of action.

Oligonucleotide drugs constitute an appealing treatment strategy from a theoretical perspective, with the capacity to alter the expression of disease-linked genes at the RNA transcript level and act synergistically alongside canonical small-molecule inhibitors [5]. Oligonucleotide drugs feature extensive target spectrum, straightforward design, long-lasting therapeutic effects and favorable safety profiles, rendering them attractive candidates for precision and personalized medical treatment [6].

*KRAS* is one of the most important oncogenic drivers in NSCLC [7]. Mutant *KRAS* promotes tumor initiation and progression through multiple downstream signaling pathways, and *KRAS*-driven lung cancer remains clinically challenging despite recent progress in *KRAS*-targeted small-molecule inhibitors. Current KRAS inhibitors are mainly effective against selected mutation subtypes, such as KRAS G12C [8,9], and their clinical benefit may be limited by resistance and incomplete response [10]. Therefore, alternative strategies capable of regulating *KRAS* expression or function remain of considerable interest. In this context, plant-derived small RNAs offer a potential source of novel regulators of KRAS and other oncogenic targets.

Increasing evidence suggests that plant-derived small RNAs can participate in cross-kingdom regulatory interactions [11]. In previous work [12], small RNAs from herbs listed in the Chinese Pharmacopoeia were systematically sequenced, generating a large resource of herb-derived small RNA candidates. In parallel, lipid nanoparticles naturally present in herbal decoctions, termed decoctosomes [12], were shown to contribute to the delivery and pharmacological activity of herbal components [13]. Inspired by these observations, synthetic mimics known as bencaosomes [12] have been developed to combine pharmacologically active small RNAs with lipid carriers for improved delivery. Building on this foundation, potential oligonucleotide drug candidates targeting various indications have been screened from multiple herbs, including pulmonary fibrosis [13], acute respiratory distress syndrome (ARDS) [14] and osteoporosis [15]. Consequently, sRNAs derived from natural herbs represent a highly potential source for novel drug development. This strategy provides a framework for transforming herbal small RNA discoveries into experimentally testable therapeutic candidates.

*Maytenus hookeri Loes.* (*M. hookeri*), primarily distributed in southern China, has been traditionally used in cancer-related applications [16]. However, the small RNA sequences from this herb and their possible anti-tumor mechanisms remain largely unknown. In the present study, we synthesized the most abundant small RNA sequences identified from *M. hookeri* and screened them in NSCLC cells, identifying MDM-sRNA-39 as a candidate with potent inhibitory activity against NSCLC cells. Our findings show that MDM-sRNA-39 suppresses NSCLC growth and downregulates *KRAS* expression. We further evaluated its effects on malignant phenotypes in vitro, formulated it with Sphingosine (d18:1) into an orally administered bencaosome for in vivo testing, thereby providing evidence that nucleic acid sequences identified from medicinal herbs may serve as a source for developing oligonucleotide therapeutics.

## 2. Results

### 2.1. MDM-sRNA-39 Suppresses Malignant Phenotypes in NSCLC Cells

**Figure 1:**
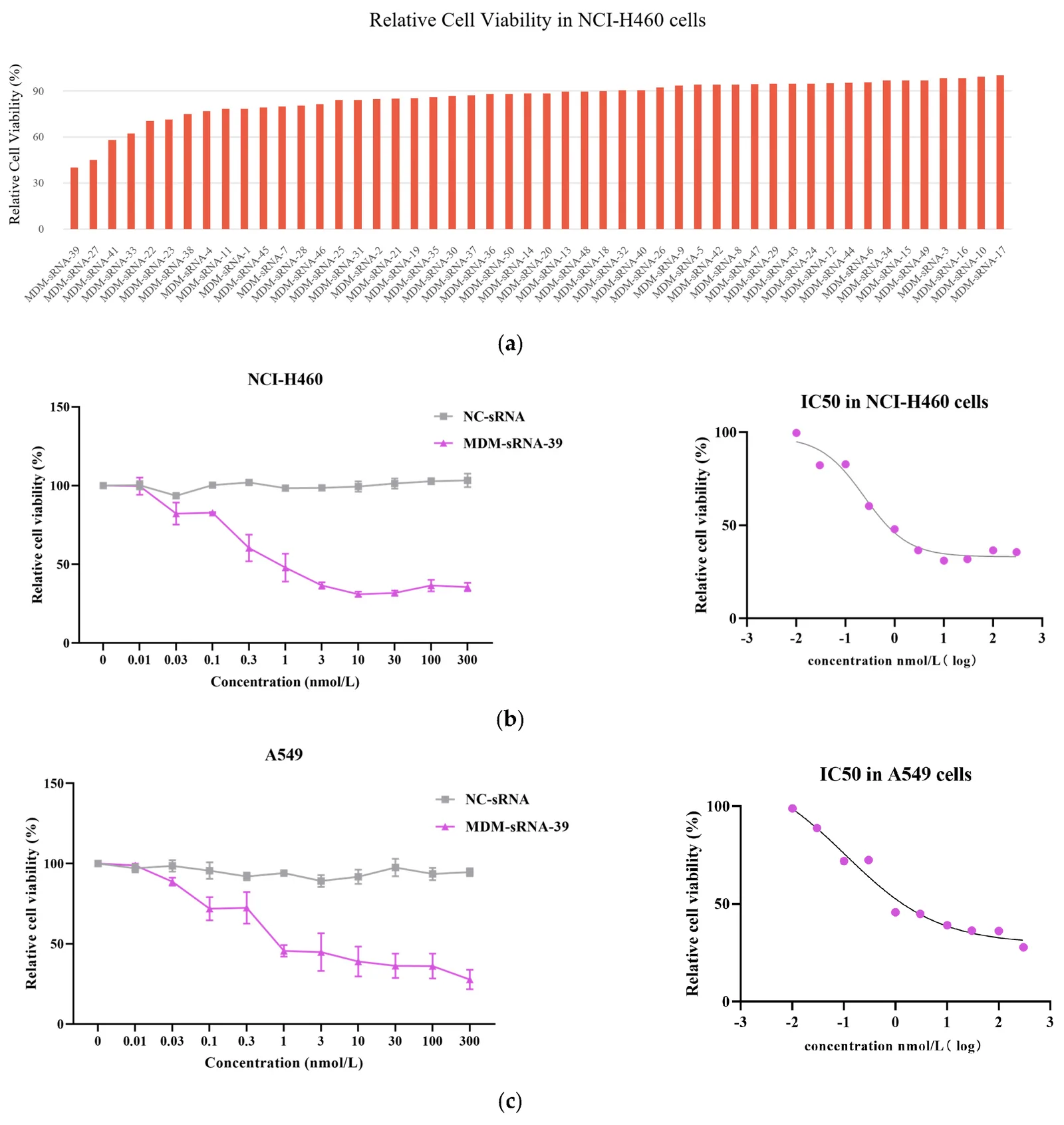

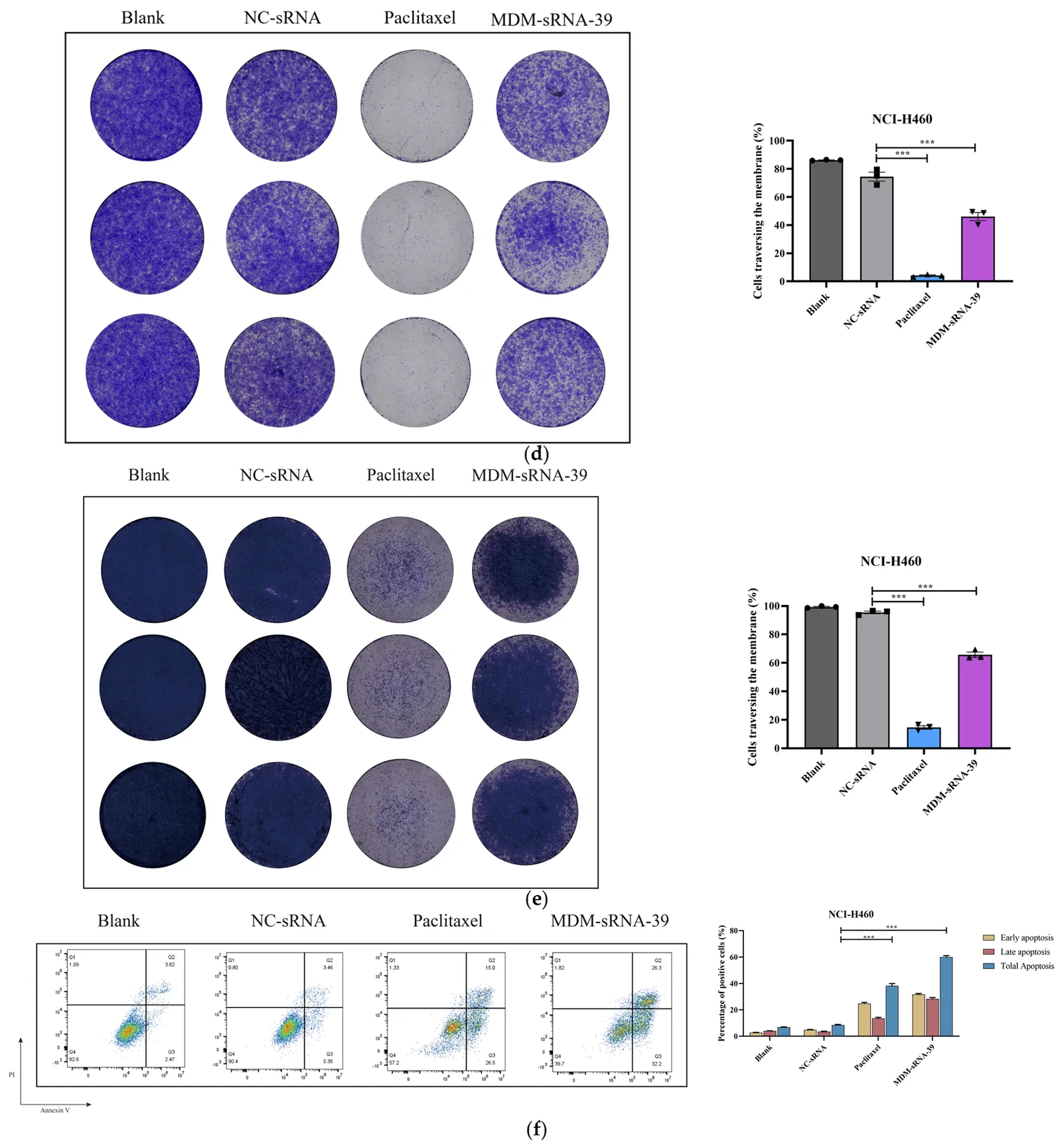
MDM-sRNA-39 suppresses malignant phenotypes in NSCLC cells. (**a**) NCI-H460 cells were transfected with *M. hookeri*-derived sRNAs (100 nmol/L). Relative cell viability (%) was measured by MTS assay 48 h post-transfection. (**b**,**c**) Representative dose–response curves for NCI-H460 (**b**) and A549 (**c**) from one of three independent experiments. Cells were transfected with a concentration gradient of sRNA (0.01–300 nmol/L). Relative cell viability (%) was measured by MTS assay 48 h post-transfection. (**d**,**e**) Percentage of cells that traversed the Transwell membrane was assessed in NCI-H460 cells after transfection (d: without Matrigel; e: with Matrigel pre-coating). (**f**) Apoptosis in NCI-H460 cells transfected with sRNA was evaluated by flow cytometry after 48 h. Data are presented as the mean ± SEM. *** *p* < 0.001.

The top 50 abundant synthetic sRNAs originating from *M. hookeri* were screened in the NCI-H460 cells using an MTS assay. Among the candidates, MDM-sRNA-39 exhibited the most potent inhibitory effect on relative cell viability (Figure 1a). The IC50 values of MDM-sRNA-39 in NCI-H460 and A549 cells were determined to be 0.47 nmol/L [95% CI: 0.10–2.15] and 0.48 [95% CI: 0.02–13.09] nmol/L, respectively, with dose-dependent inhibition of relative cell viability observed upon transfection. A representative curve is shown in Figure 1b,c; the other two independent experiments are provided in Supplementary Figure S1.

Furthermore, Transwell assays demonstrated that MDM-sRNA-39 reduces the percentage of cells that traversed the membrane in NCI-H460 (Figure 1d,e). Consistently, the wound-healing assay showed that MDM-sRNA-39 reduced wound closure compared with the NC-sRNA after 48 h (Figure S2). Flow cytometry analysis revealed an increased proportion of Annexin V/PI-positive cells, confirming that MDM-sRNA-39 promotes apoptosis in NCI-H460 cells (Figure 1f). In addition, an immortalized human bronchial epithelial cell line, BEAS-2B, and normal human fetal lung fibroblast cells, MRC-5, were treated with varying concentrations of sRNA. The results revealed low cytotoxicity of MDM-sRNA-39 toward BEAS-2B and MRC-5 cells (Figure S3).

### 2.2. MDM-sRNA-39 Suppresses KRAS Expression

**Figure 2:**
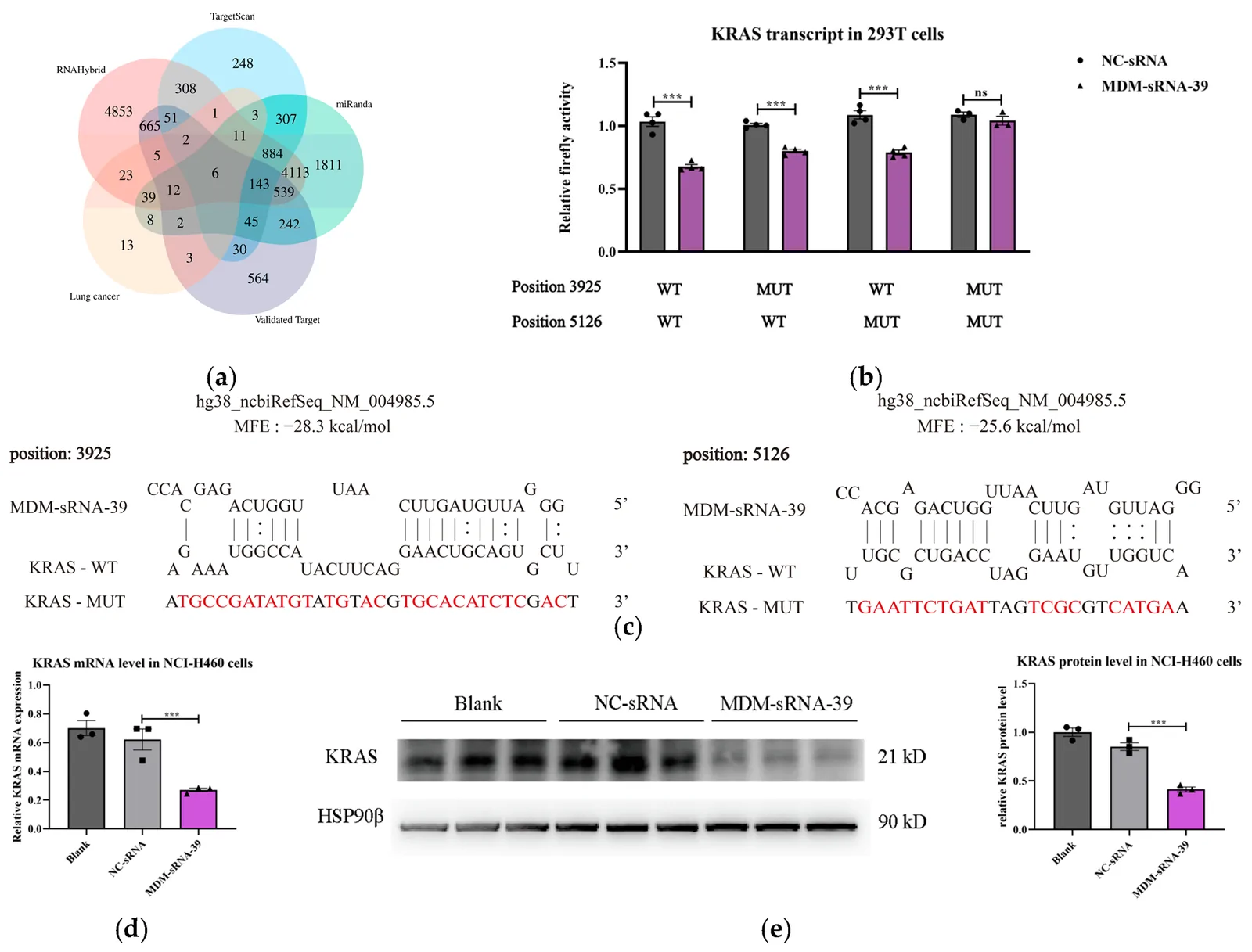
MDM-sRNA-39 suppresses *KRAS* expression. (**a**) Venn diagram of the intersection of potential targets of MDM-sRNA-39. (**b**) The relative luciferase activity was measured using a dual-luciferase reporter assay for the WT or MUT 3’UTR fragment of *KRAS*, following co-transfection with MDM-sRNA-39 or NC-sRNA. (**c**) Predicted binding position of MDM-sRNA-39 on the *KRAS* transcript fragment. Red indicates the mutated nucleotides. (**d**) *KRAS* mRNA levels after MDM-sRNA-39 transfection in NCI-H460 cells. (**e**) Western blot analysis and quantification of KRAS protein levels in NCI-H460 cells. Data are presented as the means ± SEM. ***, *p* < 0.001; ns, *p* > 0.05.

To investigate the anti-cancer mechanism of MDM-sRNA-39, we predicted its potential targets using three bioinformatics target prediction tools: RNAHybrid (MFE ≤ −30 kcal/mol), TargetScan (focusing on 7mer/8mer sites), and miRanda (MFE ≤ −18 kcal/mol). The resulting candidate genes were intersected with 128 NSCLC driver genes (IntOGen) and 2309 validated human drug targets (DrugBank), resulting in six potential targets (Figure 2a and Table 1). Preliminary RT-qPCR screening of the six candidate targets (Figure S4) identified *KRAS* as one of the most substantially downregulated genes. Given its well-established role as a critical oncogenic driver in lung cancer, *KRAS* was prioritized for experimental validation.

To experimentally validate this prediction, a dual-luciferase reporter assay was performed in HEK293T cells using a reporter construct containing the wild-type (WT) KRAS transcript. The transcript was divided into 5’UTR, CDS, and 3 overlapping fragments of 3’UTR for luciferase reporter assays. Co-transfection of MDM-sRNA-39 with reporters, the terminal fragment showed the most substantial repression (Figure S5) and contained two predicted sites (positions 3925 and 5126) (see details in Supplementary file KRAS_NM_004985.5), which were selected for further mutation analysis. When the two predicted binding sites were mutated individually, the relative luciferase activity was partially restored compared with the WT (*p* = 0.0012 and *p* = 0.0042, respectively). Moreover, when both sites were mutated simultaneously, the relative luciferase activity was comparable to that of the NC-sRNA, with no statistically significant difference (Figure 2c). These results suggest that MDM-sRNA-39 interacts with these two regions of the *KRAS* 3’UTR. RT-qPCR and Western blot analysis showed that MDM-sRNA-39 transfection markedly suppressed KRAS mRNA and protein levels in NCI-H460 cells (Figure 2d,e). Taken together, these results suggest that *KRAS* may be one of the targets of MDM-sRNA-39.

### 2.3. [Sphingosine (d18:1) + MDM-sRNA-39] Bencaosome Reduces Tumor Burden and Induces Apoptosis In Vivo

Next, the anti-tumor activity of MDM-sRNA-39 was validated in vivo. Based on our previous studies that Sphingosine (d18:1) effectively delivers sRNA in vivo [17,18], we formulated [Sphingosine (d18:1) + MDM-sRNA-39] bencaosome and characterized its basic physicochemical properties. Transmission electron microscopy (TEM) revealed the nanostructure of the bencaosome (Figure S6a). The drug-loading efficiency approached 100% when the lipid concentration increased to 0.3 mg/mL (Figure S6b). This concentration corresponds to the lipid concentration present in the bencaosome administered to the animals. The zeta potential of the bencaosome became positive compared with the lipid alone (Figure S6c). The bencaosome displayed a hydrodynamic diameter of approximately 89.0 nm (Figure S6d), an optimal size range for enhanced cellular uptake and systemic delivery.

**Figure 3:**
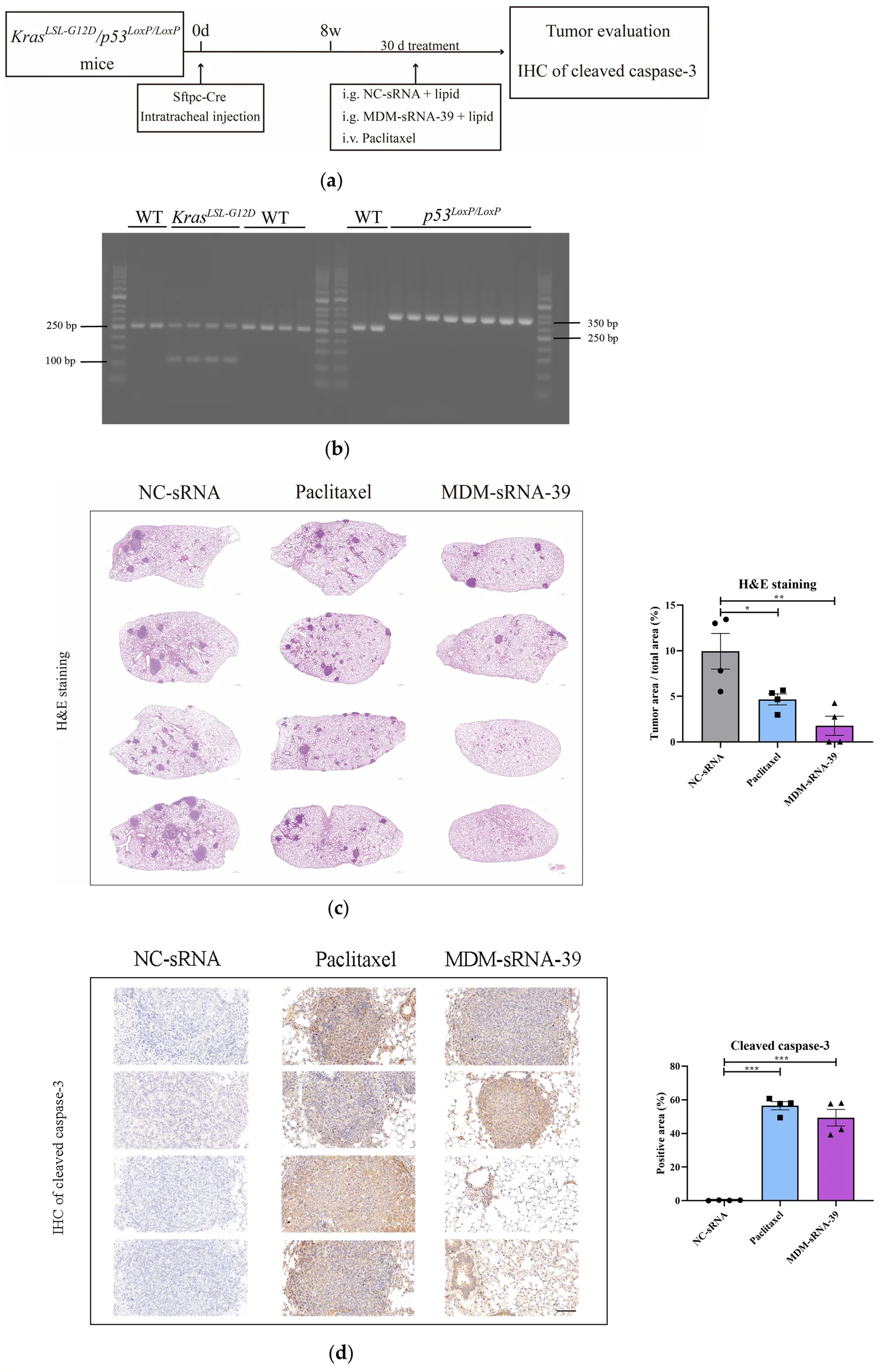
[Sphingosine (d18:1) + MDM-sRNA-39] bencaosome reduces tumor burden and induces apoptosis in vivo. (**a**) Schematic of the experimental timeline. (**b**) Genotyping of the genetically engineered mice used in the study by agarose gel electrophoresis. (**c**) Representative H&E-stained lung tissue sections from NC-sRNA (*n* = 4), paclitaxel (*n* = 4), and MDM-sRNA-39 (*n* = 4) mice. Tumor burden was quantified as the percentage of tumor area relative to total tissue area. Scale bar = 500 μm. (**d**) IHC staining for cleaved caspase-3 in lung tissue sections from KP mice. The proportion of positive area in the tumor region was analyzed. Scale bar = 100 μm. Data are presented as the means ± SEM. * *p* < 0.05; ** *p* < 0.01; *** *p* < 0.001.

A classic autochthonous lung cancer mouse model, KP (*Kras^LSL-G12D^/p53^LoxP/LoxP^*), was generated for animal experiments. Genotyping PCR was performed to confirm the genotype of KP mice. For *Kras*, the two alleles produced bands at approximately 100 bp (wild-type, WT) and 250 bp (LSL-G12D mutant), indicating a heterozygous genotype. For *p53*, the mutant allele produced a band at approximately 360 bp (Figure 3b). After 8 weeks, mice were divided into three groups: NC-sRNA, MDM-sRNA-39 and paclitaxel. The bencaosome [10 nmol sRNA + 100 μg Sphingosine (d18:1)] was administered orally to each mouse daily (Figure 3a). Paclitaxel was used as a positive control. Lung tissues of these mice were collected after 30 d of treatment, followed by staining with hematoxylin-eosin (H&E) and IHC of cleaved caspase-3. Based on the quantification of tumor burden, performed by calculating the tumor area relative to total tissue area per section from H&E staining, MDM-sRNA-39 and paclitaxel demonstrated a significant reduction compared with NC-sRNA (Figure 3c). Considering that cleaved caspase-3 is an important marker of cell apoptosis, immunohistochemical analysis revealed that MDM-sRNA-39 induced cell apoptosis in tumor regions compared with NC-sRNA, which was also observed following paclitaxel treatment (Figure 3d). Body weight was recorded throughout the treatment period (Figure S7). In summary, [Sphingosine (d18:1) + MDM-sRNA-39] bencaosome reduced the tumor burden of lung cancer and induced cell apoptosis in the KP mouse model.

## 3. Discussion

Here, we identified MDM-sRNA-39, a synthetic small RNA based on a sequence from *M. hookeri*, with potent activity against NSCLC. MDM-sRNA-39 suppressed cell viability, reduced the percentage of cells that traversed the Transwell membrane, and induced apoptosis in vitro. Additionally, tumor burden in the autochthonous lung cancer mouse model was decreased after [Sphingosine (d18:1) + MDM-sRNA-39] bencaosome treatment. These findings support the potential of MDM-sRNA-39 as an oligonucleotide drug for NSCLC.

To explore the mechanism underlying these effects, we focused on *KRAS* based on its established role as a major oncogenic driver in NSCLC. In NSCLC patients, *KRAS* is a frequently mutated oncogene, occurring in approximately 40% of lung adenocarcinomas [7]. The mutations of *KRAS*, which are the initiating genetic event [19], have a negative impact on the prognosis of NSCLC [20]. Current RAS-targeted therapeutic strategies are largely centered on inhibiting RAS protein activity, conformational switching, or effector engagement. The first clinically successful KRAS inhibitors, such as sotorasib (AMG510) [8] and adagrasib (MRTX849) [9], selectively target KRAS G12C by covalently binding to the mutant cysteine residue within the switch II pocket, preferentially stabilizing KRAS G12C in its GDP-bound inactive state. Other emerging agents follow related protein-directed principles. For example, next-generation KRAS G12C inhibitors, including divarasib [21], olomorasib [22], JDQ443 [23], garsorasib [24], and glecirasib [25] exploit the mutant cysteine residue and act through mutation-specific covalent inhibition. In contrast, KRAS G12D inhibitors such as MRTX1133 [26] use non-covalent binding to recognize a distinct mutant KRAS protein surface, whereas RAS (ON) inhibitors such as RMC-6236 [27] and RMC-6291 [28] aim to block active GTP-bound RAS signaling by interfering with effector engagement. Although these approaches have substantially advanced the druggability of RAS, they remain fundamentally dependent on specific RAS protein conformations, nucleotide states, mutation-created binding sites, or protein–protein interaction interfaces. Therefore, their therapeutic coverage may be limited by KRAS mutation subtype, adaptive pathway reactivation, and acquired resistance. The development of pan-KRAS inhibitors like JAB-23E73, which targets multiple KRAS mutants including G12A/D/V, G13D, and Q61H, highlights the clinical relevance of broad-spectrum KRAS targeting [29].

By contrast, rather than altering KRAS GTPase activity or locking a specific mutant KRAS protein in an inactive conformation, MDM-sRNA-39 acts at the transcript level by interacting with the *KRAS* 3’UTR and reducing *KRAS* mRNA and protein expression. This mechanism is distinct from protein-directed approaches, as it targets the 3’UTR rather than a particular protein pocket. MDM-sRNA-39 exhibited consistent inhibition of relative cell viability across three human NSCLC cell lines harboring distinct *KRAS* mutations (Figure S8): NCI-H460 (Q61H), A549 (G12S), and NCI-H358 (G12C). However, the cross-cell-line activity does not establish that the antiproliferative effect is mediated by *KRAS* downregulation. In the absence of rescue or epistasis experiments, we cannot conclude that KRAS is the mediator of the antitumor phenotype. In addition, sequence alignment using RNAHybrid revealed that the two predicted binding sites were relatively conserved between species (Figure S9), with MFE values of −20.1 kcal/mol and −25.8 kcal/mol for the corresponding mouse site. We acknowledge that this in silico analysis provides supportive plausibility for cross-species binding but does not constitute systematic evaluation of target-site accessibility or mouse off-targets, nor does it establish functional conservation or Kras target engagement in the KP model. Since we did not directly measure mouse *Kras* mRNA or protein levels after treatment, the in vivo anti-tumor effect observed in the KP mouse model cannot be attributed to direct suppression of mouse *Kras*. These findings are presented as supportive plausibility rather than proof of target engagement in vivo, and we have explicitly acknowledged in the Discussion that direct experimental validation of mouse Kras targeting is required. We acknowledge that systematic evaluation of mouse off-targets has not been performed and will be addressed in future studies. While other predicted targets are currently under further investigation, preliminary RT-qPCR analysis showed that several other predicted targets, including *EGFR* and *MET*, also exhibited moderate downregulation upon MDM-sRNA-39 transfection (Figure S4), suggesting that MDM-sRNA-39 may have additional targets. Their potential contributions to the anti-tumor activity warrant further investigation at the protein and functional levels.

Despite these promising results, several limitations warrant further consideration. We acknowledge that the in vitro and in vivo experiments used different RNA formats (double-stranded unmodified for in vitro; single-stranded 3′-modified for in vivo), and that we did not directly demonstrate that the modified single-stranded molecule retains KRAS-targeting activity. The in vivo efficacy and in vitro mechanistic findings are therefore presented as complementary lines of evidence rather than as proof that the same molecular species mediates both effects. Future studies directly comparing the two formats will be required to establish their functional equivalence. The Transwell assays did not fully distinguish the inhibitory effects on cell migration or invasion from the sRNA’s general cytotoxicity. Future studies at subcytotoxic doses with shorter incubation times and parallel viability measurements, normalized to viable cell numbers, will be required to further validate the specificity of these effects. We also acknowledge that the relatively small sample size (*n* = 4 per group) of mice was primarily due to limited breeding resources and the need for genotyping. Although our current analysis yielded significant results due to a remarkably large effect size, the limited sample size restricts the study’s sensitivity to only extreme effects. Sensitivity power analysis revealed that our design could only detect extremely large effects (Cohen’s f ≥ 1.069) at 80% power. Although we observed a remarkably large effect (f = 1.47) and obtained significant differences, the current findings should be interpreted with caution and warrant validation in future studies with larger sample sizes to mitigate the risk of Type II errors. Moreover, tumor burden was not assessed prior to treatment allocation, and the experiment was not blinded. These factors may introduce bias and limit the generalizability of the results. Although the observed effect size was large and statistically significant, validation in larger, blinded studies with pre-treatment tumor burden assessment will be required to further confirm the in vivo efficacy of MDM-sRNA-39. The in vivo study was designed to assess efficacy, and systematic safety evaluations (e.g., hematology, histopathology, immunotoxicity) were not performed. We also acknowledge that we did not directly measure innate immune activation markers (e.g., IFNα, ISG56) or systematically evaluate off-target effects. The chemical modification strategy of MDM-sRNA-39 was developed based on internal optimization and has not been systematically validated. Future studies with survival analysis, evaluations in immunodeficient nude mouse tumor models, and systematic evaluation of off-target effects and innate immune activation will be a crucial step for future translational development.

In conclusion, this study identifies the anti-NSCLC activity of MDM-sRNA-39, based on a sequence identified from *M. hookeri*. This study offers a novel therapeutic approach for NSCLC, expanding the repertoire of potential pharmacological interventions, and offering a valuable reference for exploring the vast traditional Chinese medicine repository to identify and develop novel, bioactive nucleic acid therapeutics.

## 4. Materials and Methods

### 4.1. Cell Lines and sRNA Transfection

The NCI-H460, A549, BEAS-2B and HEK293T cell lines were procured from the Peking Union Medical College Cell Culture Center (Beijing, China). Maintenance of NCI-H460 and BEAS-2B cells was performed in RPMI 1640 (Macgene, Beijing, China); A549 cells were cultured in F12 and HEK293T cells were cultured in DMEM, each supplemented with 1% penicillin-streptomycin and appropriate concentrations of 10% fetal bovine serum (Gibco, Waltham, MA, USA). Chemically synthesized sRNAs were obtained from GENERAL BIOL (Chuzhou, China), with sequences detailed in Table S1. For in vitro experiments, sRNA was used as an unmodified double-stranded RNA. Transfection procedures were conducted using Lipofectamine RNAiMAX reagent (Thermo Fisher Scientific, Waltham, MA, USA) with the manufacturer’s established protocols. For in vivo studies, a single-stranded RNA with 3′-terminal methoxy modifications was formulated with herbal-derived lipids for oral administration.

### 4.2. Cell Viability Assay

MTS (Promega, Madison, WI, USA) and PMS (Solarbio, Beijing, China) powders were prepared as solutions at specified concentrations and pH according to the manufacturer’s instructions. The MTS detection solution was freshly prepared by mixing Opti-MEM (Gibco, USA), MTS solution, and PMS solution at a ratio of 100:20:1 (*v*/*v*/*v*). Cells in 96-well plates were transfected with sRNAs or treated with paclitaxel (TAXOL, China). For screening, cells were transfected with sRNAs at a final concentration of 100 nmol/L. For dose–response analysis, concentrations ranging from 0.01 to 300 nmol/L were used. After 48 h, 100 µL per well of the detection solution was added, followed by incubation at 37 °C. Absorbance was subsequently recorded at 490 nm using a Synergy Neo2 microplate reader (BioTek, Winooski, VT, USA). For the initial screening (Figure 1a), relative cell viability (%) was calculated as OD490 _experimental_/OD490 _Blank_ × 100%. For other experiments on cancer cells, absorbance was recorded with background correction at 630 nm, and relative cell viability (%) was calculated as (OD490–OD630) _experimental_/(OD490–OD630) _Blank_ × 100%. “Blank” refers to untreated control cells.

IC50 values were determined by nonlinear regression using a four-parameter logistic model (log(inhibitor) vs. response-Variable slope) in GraphPad Prism 9.0 with default parameters. IC50 values are presented as geometric means with 95% confidence intervals from three independent experiments, each performed with three technical replicates per concentration. Dose–response curves covered 0.01–300 nmol/L (two experiments) or 0.1–300 nmol/L (one experiment).

### 4.3. Cytotoxicity Assay

BEAS-2B cells were transfected with sRNA or treated with paclitaxel at concentrations ranging from 0.01 to 300 nmol/L. After 48 h, the MTS assay and LDH Cytotoxicity Assay (Beyotime Biotechnology, Shanghai, China) were used for cytotoxicity; sRNA-transfected cells were normalized to RNAiMAX vehicle control, and paclitaxel-treated cells were normalized to PBS vehicle control.

MRC-5 cells were transfected with sRNA at concentrations ranging from 0.1 to 300 nmol/L. After 48 h, the MTS assay and LDH Cytotoxicity Assay (Beyotime Biotechnology, Shanghai, China) were used for cytotoxicity; cells were normalized to untreated control cells.

### 4.4. Transwell Assay and Wound-Healing Assay

3 × 10^4^ cells in serum-free medium were seeded into the upper chamber of Transwell chambers (Corning, Harrodsburg, KY, USA). 6 × 10^4^ cells were seeded into a Matrigel-coated upper chamber (pre-coated with 200–300 μg/mL Matrigel, Corning, Harrodsburg, KY, USA). After 48 h of transfection (100 nmol/L), cells that had traversed to the lower surface were fixed, stained with 0.1% crystal violet (Solarbio, Beijing, China), and viewed under DMi8 (Leica, Wetzlar, Germany). The results were measured using Image Pro.

Wound-healing assay: Cells were seeded into 12-well plates. At ≥95% confluence, scratches were made using a 10 µL pipette tip, followed by washing and incubation in serum-free medium. Images were taken by DMi6000B (Leica, Wetzlar, Germany) at 0 h and 48 h after transfection (30 nmol/L). Four fields per well were quantified using Fiji. Wound closure (%) = (area_0h_ − area_48h_)/area_0h_ × 100%.

### 4.5. Cell Apoptosis

A flow cytometer CytoFLEX (Beckman Coulter, Brea, CA, USA) was employed to detect apoptotic cells after staining with the Annexin V-FITC/PI Dead Cell Apoptosis Kit (Invitrogen, Carlsbad, CA, USA). This experiment was conducted 48 h following exposure to 100 nmol/L of NC-sRNA, MDM-sRNA-39, or paclitaxel.

### 4.6. Preparation of [Sphingosine (d18:1) + MDM-sRNA-39] Bencaosome

Sphingosine (d18:1) (Avanti Polar Lipids, Alabaster, AL, USA) was stored at −40 °C at a concentration of 10 mg/mL. Every 10 nmol MDM-sRNA-39 was mixed with 100 μg Sphingosine (d18:1) and heated at 90 °C in a water bath for 15 min.

### 4.7. Animals and Treatment

The *Kras^LSL-G12D^/p53^LoxP/LoxP^* mice (KP mice) were purchased from Jackson Laboratory. The autochthonous lung cancer mouse model was generated by intratracheal injection of a lentivirus expressing Sftpc-Cre (GENECHEM, Shanghai, China), which drives lung-specific expression of mutant *Kras* and *Trp53*. Genotype (Primer detailed in Table S2) of neonatal mice was performed using a Mouse Direct PCR Kit (Selleck, Shanghai, China), followed by agarose (LABLEAD, Beijing, China) gel electrophoresis with appropriate DNA markers (LABLEAD, Beijing, China).

For therapeutic evaluation, mice (*n* = 4 per group) were treated for 30 d with [Sphingosine (d18:1) + MDM-sRNA-39] bencaosome, administered daily via oral gavage at a dose of [10 nmol sRNA + 100 μg sphingosine (d18:1)] per mouse (administered as a fixed amount, without normalization to body weight). The NC-sRNA group received the same formulation containing NC-sRNA instead of MDM-sRNA-39. Paclitaxel was administered via tail vein injection twice weekly (total 70 nmol per week), as a positive control. Group allocation was not blinded during allocation, experiment conduct, or outcome assessment. All experiments were approved by the Animal Care and Use Committee.

### 4.8. Histopathological Examination

Harvested KP mouse lung specimens underwent paraffin embedding and sectioning post-fixation. These sections were then subjected to H&E staining for analysis of tumor burden, while cleaved caspase-3 expression (Servicebio, Wuhan, China) was detected through immunohistochemistry (IHC).

### 4.9. RT-qPCR

Total RNA was extracted using RNAiso Plus (TAKARA, Tokyo, Japan). Next, cDNA was synthesized using SuperMix (TransGen Biotech, Beijing, China). Gene expression levels (gene-specific primer sequences detailed in Table S2) were measured by RT-qPCR on a LightCycler 480 (Roche, Basel, Switzerland) using SuperMix (AQ601, TransGen Biotech, China). Relative transcript levels were calculated based on the 2^−^^ΔCt^ method.

### 4.10. Western Blot

Cells were lysed in RIPA (New Cell & Molecular Biotech, Suzhou, China), which was supplemented with protease and phosphatase inhibitor (NCM, Suzhou, China) and BeyoZonase™ Super Nuclease (Beyotime Biotechnology, Shanghai, China). Protein lysates were quantified using a BCA assay (Beyotime Biotechnology, Shanghai, China), then denatured in 5 × loading buffer (Solarbio, Beijing, China) at 100 °C for 10 min. Protein expression was assessed by standard Western blotting using SDS-PAGE. KRAS rabbit mAb (ABclonal, Wuhan, China), HSP90β rabbit mAb (Bioss, Beijing, China) and marker (Thermo Fisher Scientific, Waltham, MA, USA) were used in the Western blot.

### 4.11. Dual-Luciferase Reporter Assay

A dual-luciferase reporter assay (Promega, Madison, WI, USA) was used to test the interaction between MDM-sRNA-39 and its target genes. First, wild-type or mutant target sequences were cloned into the psiCHECK-2 vector (GENERAL BIOL, Chuzhou, China) using *Xho I* and *Not I* sites. Next, HEK293T cells were co-transfected with these plasmids (0.8 µg/mL) and sRNAs (100 nmol/L) using Lipofectamine 2000 (Thermo Fisher Scientific, Waltham, MA, USA) and RNAiMAX. After 36-48 h, luciferase activity was measured with a GloMax 96 luminometer (Promega, Madison, WI, USA). Finally, the results were evaluated by calculating the ratio of Renilla to Firefly luciferase activity.

### 4.12. Transmission Electron Microscope, Hydrodynamic Diameter Distribution and Zeta Potential Analyses

Transmission electron microscopy (TEM) images of the bencaosome were observed using a JEOL JEM-1400Plus TEM at 80 kV. The zeta potential was acquired using Zetasizer Nano ZS90 (Malvern Instrument, UK). Hydrodynamic diameter distribution was measured by PARTICLEMETRIX (ZetaView, Germany).

### 4.13. Drug-Loading Efficiency

Bencaosomes with different sRNA-to-lipid ratios were prepared, using free sRNA as a control. Quant-iT RiboGreen RNA Reagent (ThermoFisher, Waltham, MA, USA) was used with appropriate standard curves and sample dilutions. Fluorescence measurements were carried out at 485 nm excitation and 528 nm emission.

### 4.14. Target Gene Prediction for MDM-sRNA-39

Potential targets of MDM-sRNA-39 were predicted using RNAHybrid, miRanda, and TargetScan, with the intersection of results from these three methods taken for further analysis. Cancer driver genes were obtained from IntOGen (https://www.intogen.org/search, accessed on 21 June 2024). Validated human drug targets were obtained from DrugBank (https://go.drugbank.com/, accessed on 10 February 2026). Venn diagram was plotted by https://www.bioinformatics.com.cn (last accessed on 10 February 2026).

### 4.15. Statistical Analysis

Data are reported as mean ± SEM from a minimum of three independent trials. Comparisons between two groups were performed using Student‘s *t*-test. Comparisons among three or more groups were performed using one-way ANOVA followed by Dunnett’s post hoc test for comparisons against the NC-sRNA control group. *p* < 0.05 was considered statistically significant. Analysis of Western blot and the quantification of tumor burden and IHC images were carried out using NIH-developed ImageJ software (version 1.54g).

## Figures and Tables

**Table 1:** Bioinformatic prediction of MDM-sRNA-39 potential genes.

GENE	RNAHybrid (MFE *)	TargetScan	miRanda (MFE *)
*BCR*	−32.0 kcal/mol	7mer-m8	−23.19 kcal/mol
*EGFR*	−32.5 kcal/mol	7mer-1a	−22.3 kcal/mol
*KRAS*	−33.0 kcal/mol	7mer-m8	−18.75 kcal/mol
*MET*	−30.5 kcal/mol	8mer-1a	−22.2 kcal/mol
*PDGFRA*	−34.3 kcal/mol	7mer-m8	−19.39 kcal/mol
*PIK3CA*	−35.2 kcal/mol	8mer-1a	−31.1 kcal/mol

* The lowest minimal folding energy of sRNAs targets the transcript region.

## Data Availability

The original contributions presented in this study are included in the article and Supplementary Materials. Further inquiries can be directed to the corresponding author.

## Supplementary Materials

The following supporting information can be downloaded at https://www.mdpi.com/article/10.3390/ijms27177601/s1.

## Abbreviations

The following abbreviations are used in this manuscript:

NSCLC	Non-small cell lung cancer
ARDS	Acute respiratory distress syndrome
WT	wild-type
BCA	Bicinchoninic acid
DLS	Dynamic light scattering
HSP90β	Heat shock protein 90 beta
IHC	Immunohistochemistry
KP	*Kras^LSL-G12D^/p53^LoxP/LoxP^*
KRAS	Kirsten rat sarcoma viral oncogene homolog
LDH	Lactate dehydrogenase
NC	Negative control
RT-qPCR	Reverse transcription quantitative polymerase chain reaction
SEM	Standard error of the mean
TEM	Transmission electron microscopy
