# Supplementary material for: Antitumor Activity of a Synthetic Small RNA Based on a Maytenus hookeri Sequence in Non-Small Cell Lung Cancer
Source: Int J Mol Sci. 2026 Aug 25;27(17):7601. doi: 10.3390/ijms27177601 (PMC13566342; doi:10.3390/ijms27177601)
Supplement: Supplementary file 1 [file ijms-27-07601-s001.zip › Figures S1-S9.pdf]

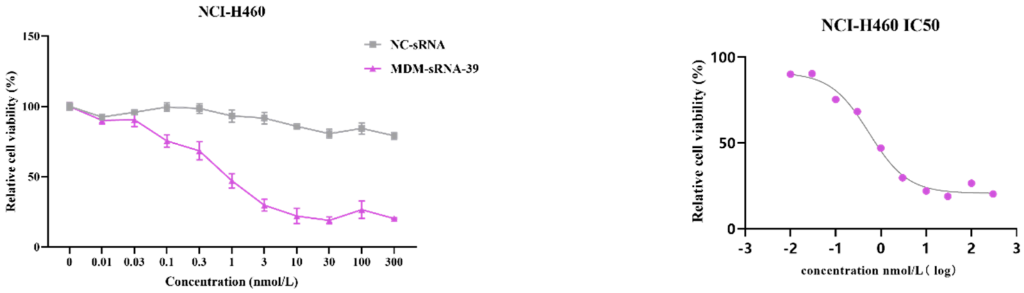

(a)

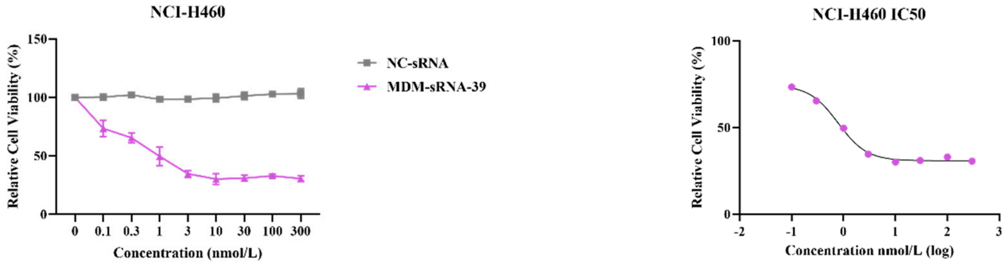

(b)

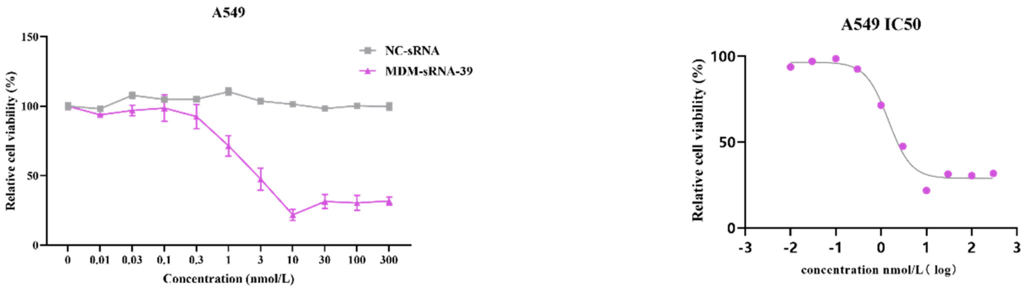

(c)

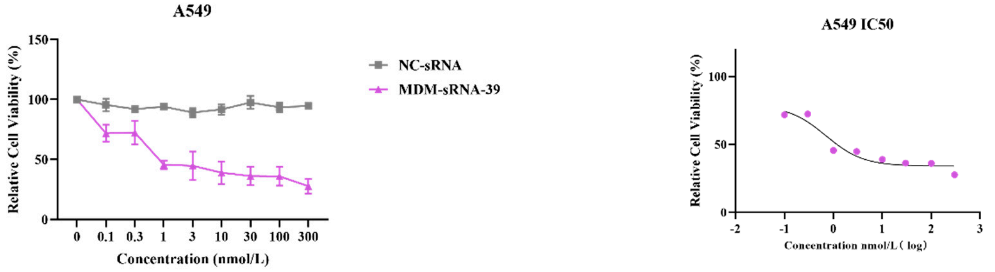

(d)

Figure S1. Independent dose-response curves for IC50 determination. (a, b) NCI-H460 and (c, d) A549 cells were transfected with a concentration gradient of sRNA. Relative cell viability (%) was measured by MTS assay 48 h post-transfection.

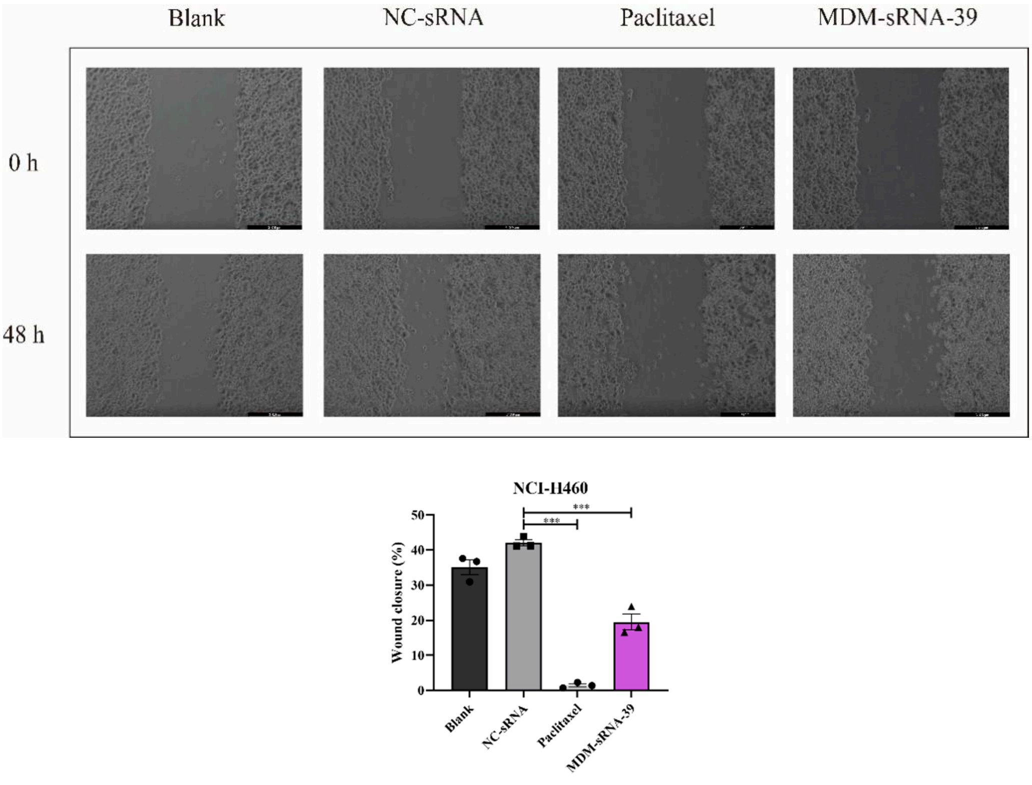

Figure S2. Effect of MDM-sRNA-39 on NCI-H460 cell in wound healing assay. Representative images of scratch wounds at 0 h and 48 h after treatment with Blank, NC-sRNA, paclitaxel, or MDM-sRNA-39 (30 nmol/L). Scale bar = 310.9 μm. Data are presented as mean ± SEM. ***P < 0.001.

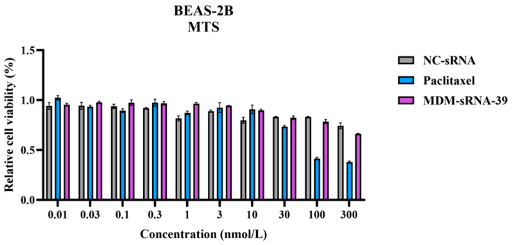

(**a**)

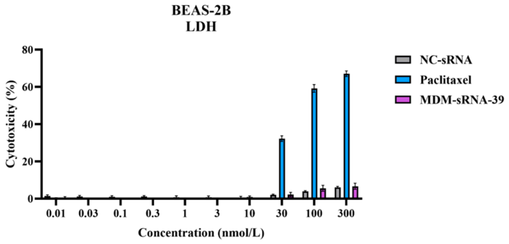

(**b**)

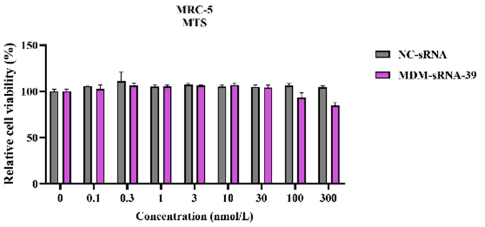

(c)

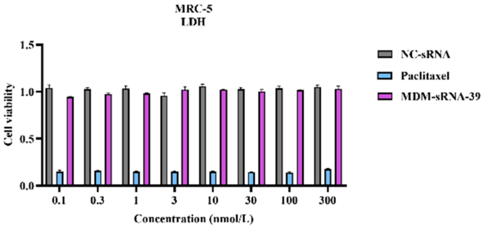

(d)

Figure S3. Cytotoxicity of MDM-sRNA-39 in BEAS-2B and MRC-5 cells. (a, b) BEAS-2B cells were transfected with a concentration gradient of MDM-sRNA-39 (0.01-300 nmol/L). (c, d) MRC-5 cells were transfected with a concentration gradient of MDM-sRNA-39 (0.1-300 nmol/L). (a, c) Relative cell viability (%) and (b, d) cytotoxicity/cell viability were assessed by MTS and LDH assays, respectively, at 48 h post-transfection.

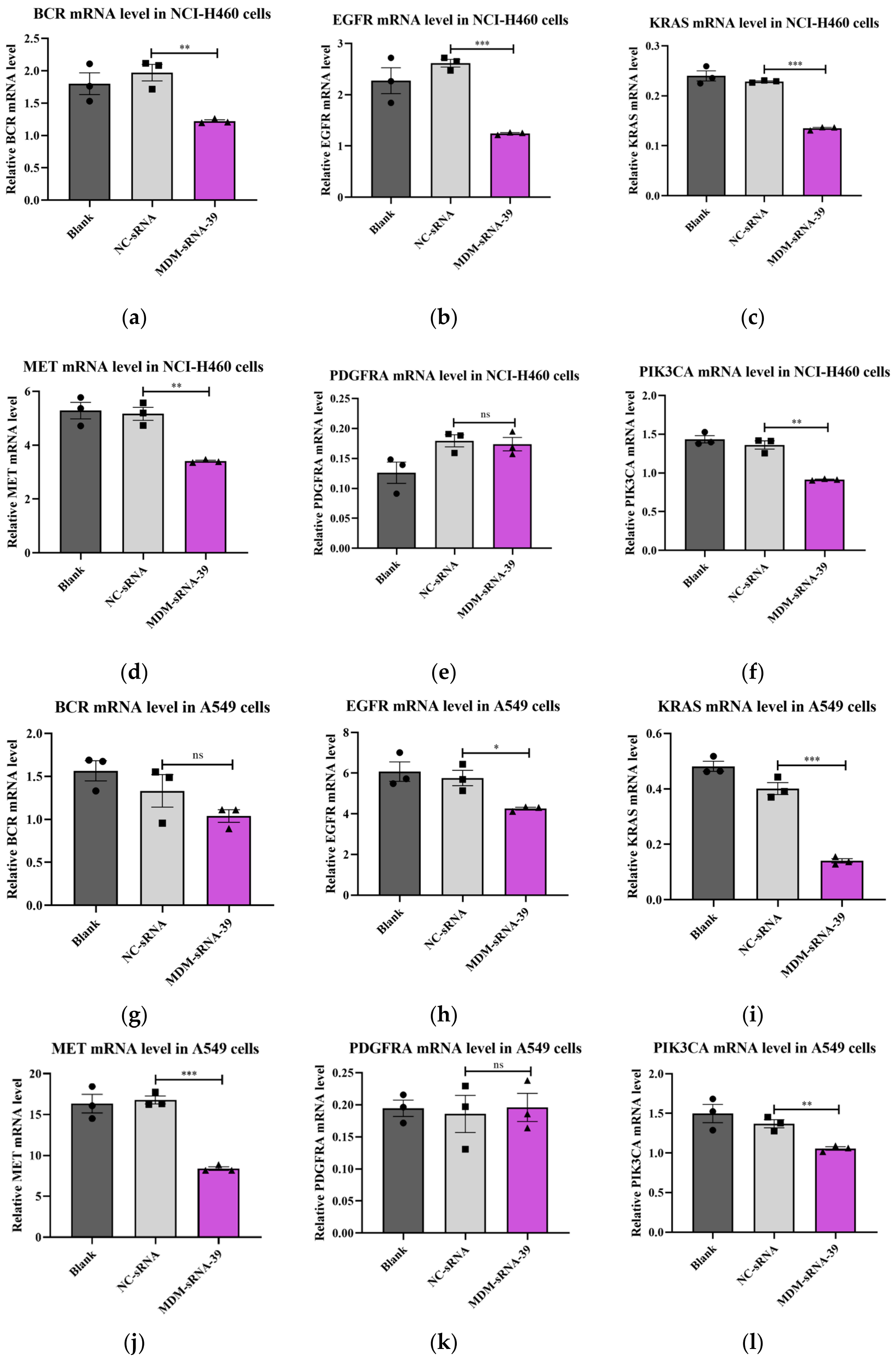

Figure S4. mRNA levels of six predicted targets in NSCLC cells after MDM-sRNA-39 transfection. (a-f) mRNA levels in NCI-H460 cells; (g-l) mRNA levels in A549 cells.

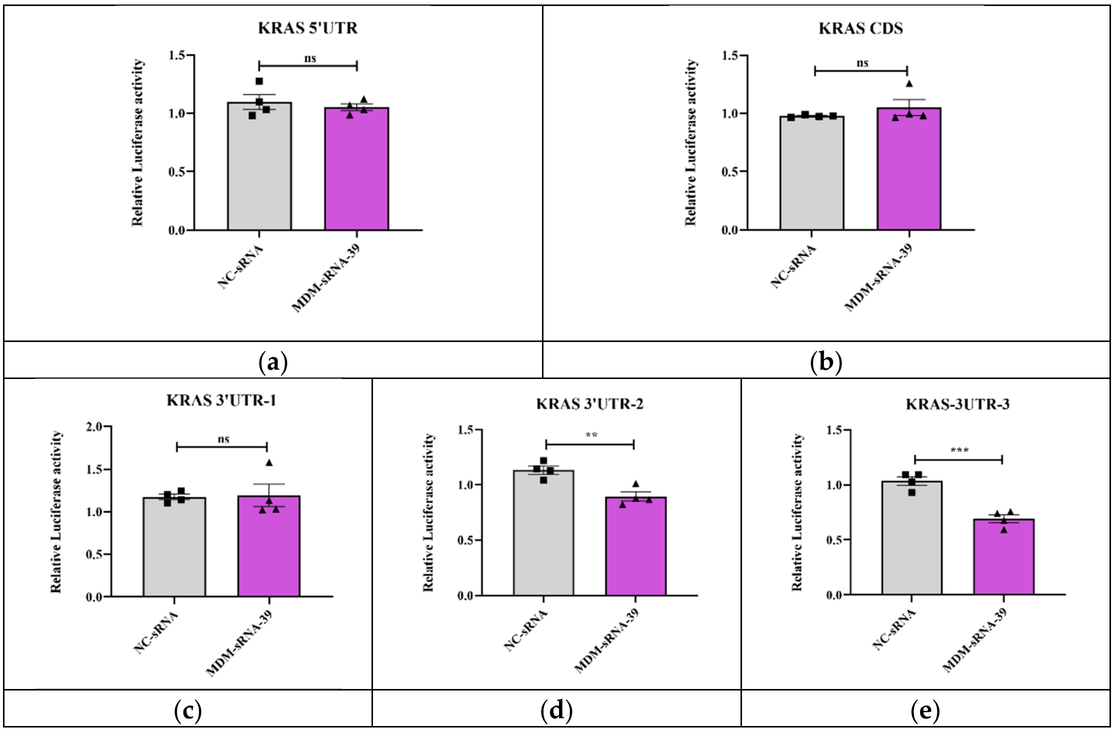

Figure S5. Dual-luciferase reporter assay of KRAS 3′UTR fragments. (a) 5′UTR, (b) CDS, and (c-e) three overlapping segments covering the 3′UTR were cloned into luciferase reporter plasmids and co-transfected with MDM-sRNA-39 or NC-sRNA into HEK293T cells. Relative luciferase activity was measured 48 h post-transfection. Data are presented as mean ±SEM. ns, $P > 0.05$, $^{**}P < 0.01$, $^{***}P < 0.001$.

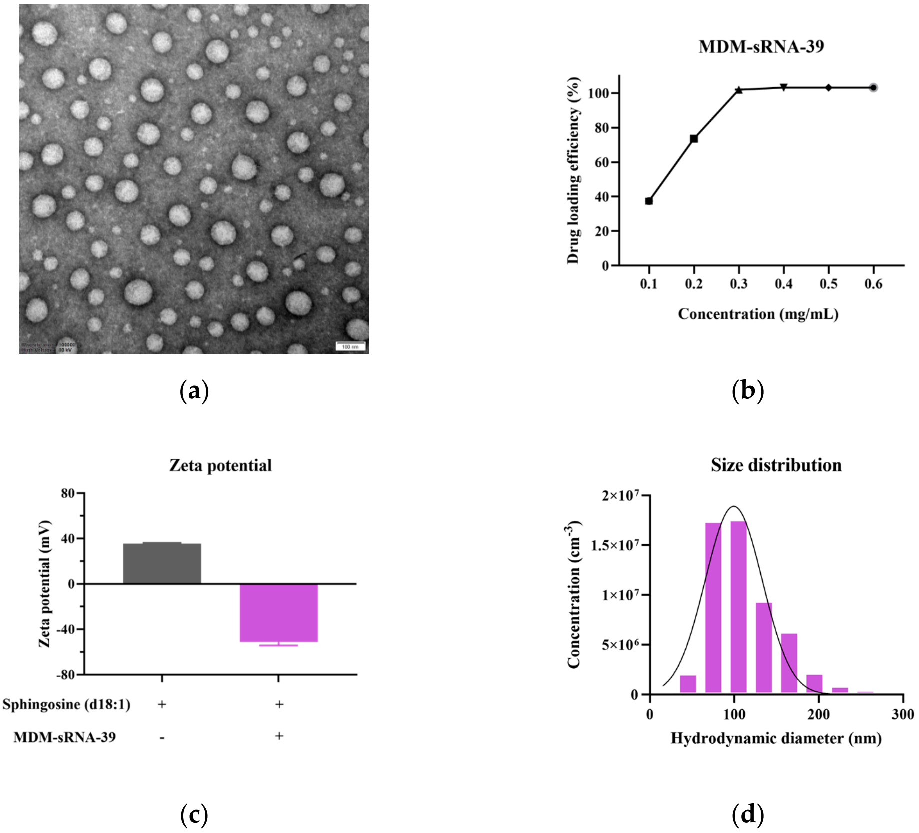

Figure S6. Characterizations of [Sphingosine (d18:1) + MDM-sRNA-39] bencaosome. (**a**) TEM image of [Sphingosine (d18:1) + MDM-sRNA-39] bencaosome nanostructure. Scale bar = 100 nm. (**b**) The drug loading efficiency of bencaosome was quantified at various lipid concentrations. (**c**) Zeta potential of Sphingosine (d18:1) with and without MDM-sRNA-39. (**d**) Hydrodynamic diameter distribution of the bencaosome, which is 100-fold diluted.

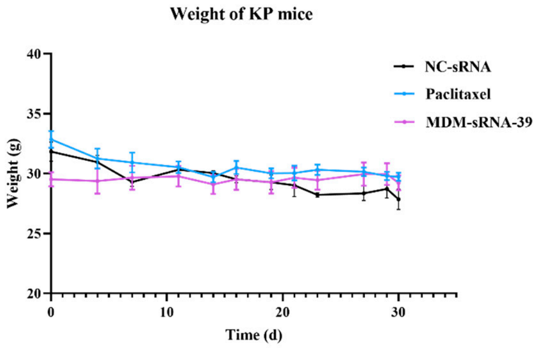

Figure S7. Body weight curves recorded during the treatment. Data are presented as mean ± SEM.

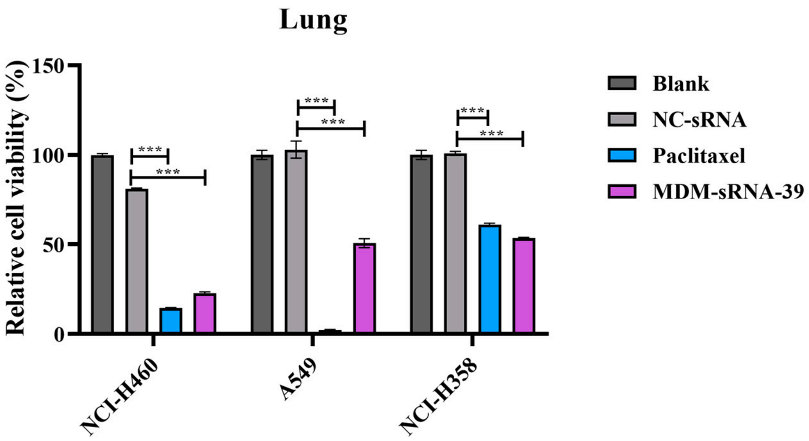

Figure S8. Relative cell viability (%) of NSCLC cell lines after 48h transfection of sRNA.

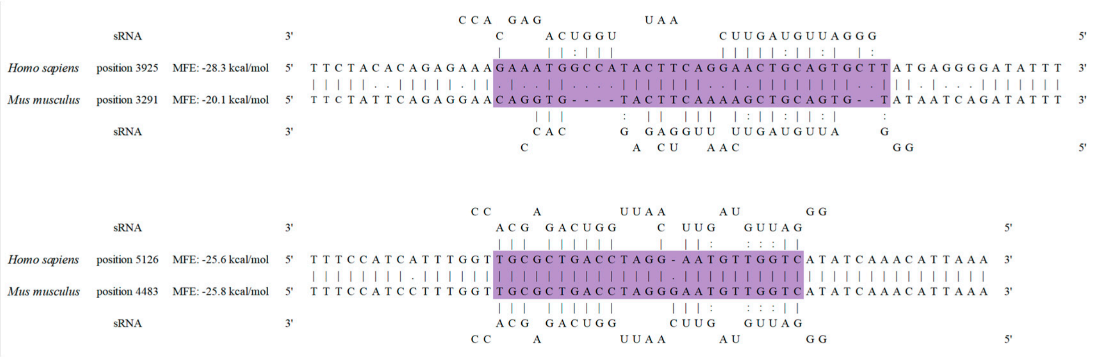

Figure S9. RNAHybrid analysis of MDM-sRNA-39 binding to human and mouse KRAS transcripts.
