# Supplementary material for: Antitumor Activity of a Synthetic Small RNA Based on a Maytenus hookeri Sequence in Non-Small Cell Lung Cancer
Source: Int J Mol Sci. 2026 Aug 25;27(17):7601. doi: 10.3390/ijms27177601 (PMC13566342; doi:10.3390/ijms27177601)
Supplement: Supplementary file 1 [file ijms-27-07601-s001.zip › KRAS_NM_004985.5.pdf]

Homo sapiens KRAS proto-oncogene, GTPase (KRAS), transcript variant b, mRNA
NCBI Reference Sequence: NM_004985.5

CDS: 191~757 (highlighted in yellow)
Overlapping fragments(highlighted in red)

1. Position 3513; MFE: -33.0 kcal/mol
2. Position 3925; MFE: -28.3 kcal/mol
3. Position 953; MFE: -27.2 kcal/mol
4. Position 2792; MFE: -27.2 kcal/mol
5. Position 138; MFE: -26.5 kcal/mol
6. Position 67; MFE: -26.1 kcal/mol
7. Position 1759; MFE: -26.0 kcal/mol
8. Position 502; MFE: -25.9 kcal/mol
9. Position 3415; MFE: -25.8 kcal/mol
10. Position 5126; MFE: -25.6 kcal/mol

CTAGGCGGCGGCCGCGGCGGCGGAGGCAGCAGCGGCGGCGGCAGTGGCGGCGG
CGAAGGTGGCGGC**GGCTCGGCCAGTACTCCCGGCCC**CCGCCATTTCGGACTGG
GAGCGAGCGCGGCGCAGGCACTGAAGGCGGC**GGCGGGGCCAGAGGCTCAGC**
GGCTCCCAGGTGCGGGAGAGAGGCCTGCTGAAAATGACTGAATATAAACTTGTG
GTAGTTGGAGCTGGTGGCGTAGGCAAGAGTGCCTTGACGATACAGCTAATTCAGA
ATCATTTTGTGGACGAATATGATCCAACAATAGAGGATTCCTACAGGAAGCAAGT
AGTAATTGATGGAGAAACCTGTCTCTTGGATATTCTCGACACAGCAGGTCAAGAG
GAGTACAGTGCAATGAGGGACCAGTACATGAGGACTGGGGAGGGCTTTCTTTGTG
TATTTGCCATAAATAATACTAAATCATTTGAAGATATTCACCATTATAGAGAACAAA
TTAAAAGAGTTAA**GGACTCTGAAGATGTACCTATGGTCCT**AGTAGGAAATAAAT
GTGATTTGCCTTCTAGAACAGTAGACACAAAACAGGCTCAGGACTTAGCAAGAA
GTTATGGAATTCCTTTTATTGAAACATCAGCAAAGACAAGACAGGGTGTTGATGAT
GCCTTCTATACATTAGTTCGAGAAATTCGAAAACATAAAGAAAAGATGAGCAAAG
ATGGTAAAAAGAAGAAAAAGAAGTCAAAGACAAAGTGTGTAATTATGTAAATAC
AATTTGTACTTTTTTCTTAAGGCATACTAGTACAAGTGGTAATTTTTGTACATTACAC
TAAATTATTAGCATTTGTTTTAGCATTACCTAATTTTTTTCCTGCTCCATGCAGACTG
TTAGCTTTTACCTTAAATGCTTATTTTAAAATGACAGTGGAAGTTTTTTTTTCCTCTA
AGTGCCAGTATTCCCAGA**GTTTTGGTTTTTGAACTAGCAATGCCT**GTGAAAAAG
AAACTGAATACCTAAGATTTCTGTCTTGGGGCTTTTGGTGCATGCAGTTGATTACTT
CTTATTTTTCTTACCAATTGTGAATGTTGGTGTGAAACAAATTAATGAAGCTTTTGA
ATCATCCCTATTCTGTGTTTTATCTAGTCACATAAATGGATTAATTACTAATTTCAGT
TGAGACCTTCTAATTGGTTTTTACTGAAACATTGAGGGAACACAAATTTATGGGCT
TCCTGATGATGATTCTTCTAGGCATCATGTCCTATAGTTTGTCATCCCTGATGAATGT
AAAGTTACACTGTTCACAAAGGTTTTGTCTCCTTTCCACTGCTATTAGTCATGGTCA
CTCTCCCCAAAATATTATATTTTTTCTATAAAAAGAAAAAAATGGAAAAAAATTAC
AAGGCAATGGAAACTATTATAAGGCCATTTCCTTTTCACATTAGATAAATTACTATA

AAGACTCCTAATAGCTTTTCCTGTTAAGGCAGACCCAGTATGAAATGGGGATTATT
ATAGCAACCATTTTGGGGCTATATTTACATGCTACTAAATTTTTATAATAATTGAAA
AGATTTTAACAAGTATAAAAAATTCTCATAGGAATTAAATGTAGTCTCCCTGTGTC
AGACTGCTCTTTCATAGTATAACTTTAAATCTTTTCTTCAACTTGAGTCTTTGAAGA
TAGTTTTAATTCTGCTTGTGACATTAAAAGATTATTTGGGCCAGTTATAGCTTATTAG
GTGTTGAAGAGACCAAGGTTGCAAGGCCAGGCCCTGTGTGAACCTTTGAGCTT
TCATAGAGAGTTTCACAGCATGGACTGTGTCCCCACGGTCATCCAGTGTTGTCA
TGCATTGGTTAGTCAAAATGGGGAGGGACTAGGGCAGTTTGGATAGCTCAACAAG
ATACAATCTCACTCTGTGGTGGTCCTGCTGACAAATCAAGAGCATTGCTTTTGTTT
CTTAAGAAAACAAACTCTTTTTTAAAAATTACTTTTAAATATTAACTCAAAAGTTG
AGATTTTGGGGTGGTGGTGTGCCAAGACATTAATTTTTTTTTTAAACAATGAAGTG
AAAAAGTTTTACAATCTCTAGGTTTGGCTAGTTCTCTTAACACTGGTTAAATTAAC
ATTGCATAAACACTTTTCAAGTCTGATCCATATTTAATAATGCTTTAAAATAAAAAT
AAAAACAATCCTTTTGATAAATTTAAAATGTTACTTATTTTAAAATAAATGAAGTG
AGATGGCATGGTGAGGTGAAAGTATCACTGGACTAGGAAGAAGGTGACTTAGGT
TCTAGATAGGTGTCTTTTAGGACTCTGATTTTGAGGACATCACTTACTATCCATTTC
TTCATGTTAAAAGAAGTCATCTCAAACTCTTAGTTTTTTTTTTTTTACAACTATGTAAT
TTATATTCCATTTACATAAGGATACACTTATTTGTCAAGCTCAGCACAATCTGTAAA
TTTTTAACCTATGTTACACCATCTTCAGTGCCAGTCTTGGGCAAAATTGTGCAAGA
GGTGAAGTTTATATTTGAATATCCATTCTCGTTTTAGGACTCTTCTTCCATATTAGTG
TCATCTTGCCTCCCTACCTTCCACATGCCCCATGACTTGATGCAGTTTTAATACTTG
TAATTCCCCTAACCATAAGATTTACTGCTGCTGTGGATATCTCCATGAAGTTTTCCC
ACTGAGTCACATCAGAAATGCCCTACATCTTATTTCCTCAGGGCTCAAGAGAATCT
GACAGATACCATAAAGGGATTTGACCTAATCACTAATTTTCAGGTGGTGGCTGATG
CTTTGAACATCTCTTTGCTGCCCAATCCATTAGCGACAGTAGGATTTTTCAAACC
TGGTATGAATAGACAGAACCCTATCCAGTGGAAGGAGAATTTAATAAAGATAGTG
CTGAAAGAATTCCTTAGGTAATCTATAACTAGGACTACTCCTGGTAACAGTAATAC
ATTCCATTGTTTTAGTAACCAGAAATCTTCATGCAATGAAAAATACTTTAATTCATG
AAGCTTACTTTTTTTTTTTGGTGTCAGAGTCTCGCTCTTGTCACCCAGGCTGGAATG
CAGTGGCGCCATCTCAGCTCACTGCAACCTCCATCTCCCAGGTTCAAGCGATTCTC
GTGCCTCGGCCTCCTGAGTAGCTGGGATTACAGGCGTGTGCCACTACACTCAACT
AATTTTTGTATTTTTAGGAGAGACGGGGTTTCACCCTGTTGGCCAGGCTGGTCTCG
AACTCCTGACCTCAAGTGATTCACCCACCTTGGCCTCATAAACCTGTTTTGCAGAA
CTCATTTATTCAGCAAATATTTATTGAGTGCCTACCAGATGCCAGTCACCACACAA
GGCACTGGGTATATGGTATCCCCAAACAAGAGACATAATCCCGGTCCTTAGGTAG
TGCTAGTGTGGTCTGTAATATCTTACTAAGGCCTTTGGTATACGACCCAGAGAT
AACACGATGCGTATTTTAGTTTTGCAAAGAAGGGGTTTGGTCTCTGTGCCAGCTCT
ATAATTGTTTTGCTACGATTCCACTGAAACTCTTCGATCAAGCTACTTTATGTAAA
TCACTTCATTGTTTTAAAGGAATAAACTTGATTATATTGTTTTTTTATTTGGCATAAC
TGTGATTCTTTTAGGACAATTACTGTACACATTAAGGTGTATGTCAGATATTCATATT
GACCCAAATGTGTAATATTCCAGTTTTCTCTGCATAAGTAATTAAAATATACTTAAA
AATTAATAGTTTTATCTGGGTACAAATAAACAGGTGCCTGAACTAGTTCACAGACA
AGGAAACTTCTATGTAAAAATCACTATGATTTCTGAATTGCTATGTGAAACTACAG
ATCTTTGGAACACTGTTTAGGTAGGGTGTTAAGACTTACACAGTACCTCGTTTCTA

CACAGAGAA**AGAAATGGCCATACTTCAGGAACTGCAGTGCTT**ATGAGGGGATA
TTTAGGCCTCTTGAATTTTTGATGTAGATGGGCATTTTTTTAAGGTAGTGGTTAATTA
CCTTTATGTGAACTTTGAATGGTTTAACAAAAGATTTGTTTTTGTAGAGATTTTAAA
GGGGGAGAATTCTAGAAATAAATGTTACCTAATTATTACAGCCTTAAAGACAAAA
ATCCTTGTTGAAGTTTTTTTAAAAAAAGCTAAATTACATAGACTTAGGCATTAACAT
GTTTGTGGAAGAATATAGCAGACGTATATTGTATCATTTGAGTGAATGTTCCCAAG
TAGGCATTCTAGGCTCTATTTAACTGAGTCACACTGCATAGGAATTTAGAACCTAA
CTTTTATAGGTTATCAAAACTGTTGTCACCATTGCACAATTTTGTCCTAATATATACA
TAGAAACTTTGTGGGGCATGTTAAGTTACAGTTTGCACAAGTTCATCTCATTTGTAT
TCCATTGATTTTTTTTTTCTTCTAAACATTTTTTCTTCAAACAGTATATAACTTTTTTT
AGGGGATTTTTTTTTAGACAGCAAAAACTATCTGAAGATTTCCATTTGTCAAAAAG
TAATGATTTCTTGATAATTGTGTAGTAATGTTTTTTAGAACCCAGCAGTTACCTTAA
AGCTGAATTTATATTTAGTAACTTCTGTGTTAATACTGGATAGCATGAATTCTGCATT
GAGAAACTGAATAGCTGTCATAAAATGAAACTTTCTTTCTAAAGAAAGATACTCA
CATGAGTTCTTGAAGAATAGTCATAACTAGATTAAGATCTGTGTTTTAGTTTAATAG
TTTGAAGTGCCTGTTTGGGATAATGATAGGTAATTTAGATGAATTTAGGGGAAAAA
AAAGTTATCTGCAGATATGTTGAGGGCCCATCTCTCCCCCCACACCCCCACAGAG
CTAACTGGGTTACAGTGTTTTATCCGAAAGTTTCCAATTCCACTGTCTTGTGTTTTC
ATGTTGAAAATACTTTTGCATTTTTCCTTTGAGTGCCAATTTCTTACTAGTACTATTT
CTTAATGTAACATGTTTACCTGGAATGTATTTTAACTATTTTTGTATAGTGTAAACTG
AAACATGCACATTTTGTACATTGTGCTTTCTTTTGTGGGACATATGCAGTGTGATCC
AGTTGTTTTCCATCATTTGG**TTGCGCTGACCTAGGAATGTTGGTCA**TATCAAACA
TTAAAAATGACCACTCTTTTAATTGAAATTAACTTTTAAATGTTTATAGGAGTATGT
GCTGTGAAGTGATCTAAAATTTGTAATATTTTTGTCATGAACTGTACTACTCCTAAT
TATTGTAATGTAATAAAAATAGTTACAGTGAC
