# Supplementary material for: Antitumor Activity of a Synthetic Small RNA Based on a Maytenus hookeri Sequence in Non-Small Cell Lung Cancer
Source: Int J Mol Sci. 2026 Aug 25;27(17):7601. doi: 10.3390/ijms27177601 (PMC13566342; doi:10.3390/ijms27177601)
Supplement: Supplementary file 1 [file ijms-27-07601-s001.zip › Tables S1 and S2.pdf]

Table S1. Sequences of top 50 abundant sRNAs derived from *Maytenus hookeri Loes.*

sRNA name	sRNA sequence
MDM-sRNA-1	UCCGUUGUCGUCCAGCGGUUAGGAUAUCUGG
MDM-sRNA-2	AGUUACUAAUUCAUGAUCUGGC
MDM-sRNA-3	CAGCCCUAUGUCGCUCCGAUUCGU
MDM-sRNA-4	GCGUCUGUAGUCCAACGGUUAGGAUAAUUGC
MDM-sRNA-5	UCCGUUGUCGUCCAGCGGUUAGGAUAUCUG
MDM-sRNA-6	UAUGUCGCUCCGAUUCGU
MDM-sRNA-7	GCGUCUGUAGUCCAACGGUUAGGAUA
MDM-sRNA-8	CCCUAUGUCGCUCCGAUUCGU
MDM-sRNA-9	GGCGGAUGUAGCCAAGUGGA
MDM-sRNA-10	GCGCCUGUAGCUCAGUGGA
MDM-sRNA-11	AGGGCACGUCUGCCUGGGUGUCACGU
MDM-sRNA-12	CUGGGAAGUCCUCGUGUUGCACCCUC
MDM-sRNA-13	UCCGUUGUCGUCCAGCGGUUAGGAUAUCU
MDM-sRNA-14	CUGGGAAGUCCUCGUGUUGCACCCGC
MDM-sRNA-15	UGAAACGACUCUCGGCAACGGAUA
MDM-sRNA-16	CUAUGUCGCUCCGAUUCGU
MDM-sRNA-17	GAAACGACUCUCGGCAACGGAUA
MDM-sRNA-18	CCUAUGUCGCUCCGAUUCGU
MDM-sRNA-19	CACGUCUGCCUGGGUGUCACGU
MDM-sRNA-20	GGGGAUGUAGCUCAGAUGGUAGAGC
MDM-sRNA-21	CCGAGGGCACGUCUGCCUGGGUGUCACGU
MDM-sRNA-22	AUUCGGUCGGGAACUCAAAGGAGACUGCC
MDM-sRNA-23	UCCUCUGUAGUUCAGUCGGUAGAACGGC
MDM-sRNA-24	GGGGAUGUAGCUCAGAUGGU
MDM-sRNA-25	UACCUGGUUGAUCCUGCC
MDM-sRNA-26	AUUCAGCCCUAUGUCGCUCCGAUUCGU
MDM-sRNA-27	UCCAUUGUCGUCUAGUCCGGUUUAGGAUA
MDM-sRNA-28	GGGGAUGUAGCUCAGAUGG
MDM-sRNA-29	UGAAACGACUCUCGGCAACG
MDM-sRNA-30	UGAAACGACUCUCGGCAACGGA
MDM-sRNA-31	UUCAGCCCUAUGUCGCUCCGAUUCGU
MDM-sRNA-32	GGGGAUGUAGCUCAGAUGGUA
MDM-sRNA-33	GAAACGACUCUCGGCAACGGAUAUC
MDM-sRNA-34	GGGCCUGUAGCUCAGAGGA
MDM-sRNA-35	CGGCGGACUGCUCGAGCUGCU
MDM-sRNA-36	UGAAACGACUCUCGGCAACGG
MDM-sRNA-37	CCUGCGGAAGGAUCAUUG
MDM-sRNA-38	CUUGAGUUCGACUCUCAACGAGAGCACCA
MDM-sRNA-39	GGGAUUGUAGUUCAAUUGGUCAGAGCACC
MDM-sRNA-40	GGGGAUGUAGCUCAGAUG
MDM-sRNA-41	UGAAACGACUCUCGGCAACGGAUAUC
MDM-sRNA-42	AGCCCUAUGUCGCUCCGAUUCGU
MDM-sRNA-43	CUGGGAAGUCCUCGUGUUGCACCCGCC
MDM-sRNA-44	GACCGCAUAGCGCAGUGGA
MDM-sRNA-45	AGUUACUAAUUCAUGAUCUGGU
MDM-sRNA-46	AACGACUCUCGGCAACGGAUA
MDM-sRNA-47	UCCGUUGUAGUCUAGUUGG
MDM-sRNA-48	GAAACGACUCUCGGCAACGGA
MDM-sRNA-49	UCAGCCCUAUGUCGCUCCGAUUCGU
MDM-sRNA-50	CGUCUGCCUGGGUGUCACGU
NC-sRNA	UUCUCCGAACGUGUCACGUTT

Table S2. Sequences of PCR primers

Name	Primer	Sequence (5'-3')
Mus P53	Forward	GGTTAAACCCAGCTTGACCA
Mus P53	Reverse	GGAGGCAGAGACAGTTGGAG
Mus Kras	Forward (WT)	TGTCTTTCCCCAGCACAGT
Mus Kras	Forward (Mut)	GCAGGTCGAGGGACCTAATA
Mus Kras	Reverse (Common)	CTGCATAGTACGCTATACCCTGT
HUMAN TBP	Forward	GCCCGAAACGCCGAATATAA
HUMAN TBP	Reverse	TGCCAGTCTGGACTGTTCTT
HUMAN KRAS	Forward	CAGTAGACACAAAACAGGCTCAG
HUMAN KRAS	Reverse	TGTCGGATCTCCCTCACCAATG
